# Prevalence, predictors, and clinical impact of vitamin D deficiency on 12-month outcomes in hip fracture patients

**DOI:** 10.1007/s40618-026-02952-x

**Published:** 2026-06-27

**Authors:** Gaetano Paride Arcidiacono, Marco Onofrio Torres, Chiara Ceolin, Giovanni Tripepi, Valentina Camozzi, Anna Bertocco, Mor Peleg Falb, Martin Diogo, Francesca Guidolin, Alberta Cecchinato, Giacomo Voltan, Michela Ferrarese, Maria Grazia Rodà, Elisa Pala, Andrea Angelini, Deris Gianni Boemo, Maria Vittoria Nesoti, Elena Campello, Pietro Ruggieri, Maria Fusaro, Salvatore Minisola, Paolo Simioni, Giuseppe Sergi, Sandro Giannini, Marina De Rui, Stefania Sella

**Affiliations:** 1 Department of Medicine, University of Padova, Padova, Italy; 2 Department of Medicine, Clinica Medica 1, Azienda Ospedale-Università Padova, Padova, Italy; 3 Geriatrics Division, Department of Medicine, Azienda Ospedale-Università Padova, Padova, Italy; 4 Institute of Clinical Physiology (IFC), National Research Council (CNR), Reggio Calabria, Italy; 5 Endocrinology Unit, Department of Medicine, Azienda Ospedale-Università Padova, Padova, Italy; 6 Department of Orthopedics and Orthopedic Oncology, D.I.S.C.O.G. Department, University of Padova, Padova, Italy; 7 Department of Directional Hospital Management, Azienda Ospedale-Università Padova, Padova, Italy; 8 Institute of Clinical Physiology (IFC), National Research Council (CNR), Pisa, Italy; 9 Sapienza University of Rome, Rome, Italy; 10 Metabolic Bone Disease Unit, Azienda Ospedale-Università Padova, Padova, Italy

**Keywords:** Hip fracture, Vitamin D deficiency, Osteoporosis, Fracture Liaison Service, Mortality

## Abstract

**Purpose:**

Vitamin D deficiency (VDD) is common in older adults with osteoporosis, but its prevalence, determinants, and clinical impact in hip fracture (HF) patients remain incompletely defined. This study investigated the prevalence of VDD, associated factors, and 12-month outcomes in HF patients managed within a Fracture Liaison Service program.

**Methods:**

In this prospective cohort study, 934 patients hospitalized for HF were enrolled between March 2023 and March 2025. VDD was defined as serum 25-hydroxyvitamin D [25-(OH)D] < 50 nmol/L. Clinical characteristics, laboratory parameters, and 12-month outcomes were assessed.

**Results:**

Overall VDD prevalence was 53.6%, with a median 25-(OH)D concentration of 22 nmol/L among deficient patients. Independent predictors of VDD were male sex (*p* < 0.001), diabetes mellitus (*p* = 0.003), dementia (*p* = 0.013), and active smoking (*p* = 0.045), while prior vitamin D supplementation was negatively associated (*p* < 0.001). Decision tree analysis showed VDD prevalence ranging from 17.2% to 86.3% across patient phenotypes. Patients with VDD showed higher levels of bone turnover markers. In unadjusted analyses, VDD was associated with higher 12-month mortality (HR 1.65, 95% CI 1.12–2.42, *p* = 0.010), an association attenuated and no longer significant after adjustment for age, sex, and comorbidity burden (HR 1.31, 95% CI 0.88–1.96, *p* = 0.188). No associations were observed between VDD and rehospitalization or refracture rates.

**Conclusion:**

VDD is highly prevalent in HF patients. Although associated with higher mortality in unadjusted analyses, this relationship appears largely explained by comorbidity burden. These findings highlight the importance of recognizing VDD and implementing targeted vitamin D supplementation strategies in high-risk HF populations.

**Supplementary Information:**

The online version contains supplementary material available at https://doi.org/10.1007/s40618-026-02952-x.

## Introduction

Fragility fractures (FF) resulting from low-energy trauma represent a major complication of osteoporosis [1]. Hip fractures (HF) are the most severe type of FF, with approximately 5% mortality at one month and 20% at one year [2]. They also lead to significant morbidity: up to 30% of patients develop permanent disability, 40% lose independent mobility, and 80% require assistance with daily living activities [2]. As the population ages, the absolute number of HF in Europe is expected to double by 2050 [3]. Anti-fracture treatment plays a crucial role in preventing subsequent fractures, with effective drugs available to reduce refracture risk and FF-related mortality [4]. However, the osteoporosis ‘treatment gap’ remains a major challenge, particularly among patients who have experienced a FF, with over 80% of eligible patients not receiving appropriate anti-fracture therapy within one year of the event [5]. In response, various post-fracture care programs, such as the Fracture Liaison Service (FLS), have been developed to prevent recurrent fractures in both hospitalized and outpatient settings. FLS represents a coordinated care model designed to systematically identify, assess, and manage patients with FF, ensuring they receive appropriate anti-fracture treatment [6]. These programs have proven effective in increasing treatment initiation rates and reducing the risk of subsequent fractures [7]. Vitamin D deficiency (VDD) represents a potential contributor to skeletal fragility. While the 2024 revision of the Endocrine Society guidelines for Vitamin D for the Prevention of Disease does not propose specific thresholds for 25-hydroxyvitamin D [25(OH)D] adequacy, levels below 50 nmol/L are generally considered indicative of VDD and appropriate for intervention [8, 9]. VDD is highly prevalent among certain populations, particularly older adults and individuals with osteoporosis [10]. VDD is well established to have adverse skeletal consequences, including accelerated bone turnover, bone loss, and increased osteoporotic fracture risk [11]. Furthermore, low vitamin D levels can diminish the efficacy of various anti-resorptive drugs [12, 13]. In HF patients specifically, VDD has been associated with increased fracture risk, higher mortality, reduced quality of life, and impaired functional recovery [14, 15], although the independent contribution of VDD versus associated comorbidities to these outcomes still requires further clarification. Given these premises, this study aims to investigate the prevalence of VDD in patients hospitalized for HF, identify key demographic and clinical factors associated with its presence, and evaluate its relationship with bone health as well as 12-month clinical outcomes.

## Patients and methods

### Study population

We conducted a prospective cohort study enrolling patients aged 50 years and older who were acutely admitted with HF at the University Hospital of Padua (Italy), within the Hip-Padua Osteosarcopenia (Hip-POS) FLS program. The full structure of Hip-POS has been previously described [16]. This study was conducted and reported according to the STROBE (Strengthening the Reporting of Observational Studies in Epidemiology) guidelines for cohort studies [17]. For this analysis, we included patients assessed during their in-hospital stay between March 2023 and March 2025. Subjects with traumatic or pathological fractures (i.e., primary or secondary bone tumors, Paget’s bone disease) were excluded. After providing informed consent, patients were clinically examined, evaluated for their risk factors for skeletal fragility and underwent laboratory tests related to mineral metabolism. Among the enrolled patients, those whose serum 25(OH)D levels were not available at the time of hospital admission were excluded.

### Data collection

We reviewed computerized medical records from the in-hospital stay period, from which the following data were extracted: demographic and anthropometric characteristics; comorbidities, including the Charlson Comorbidity Index (CCI) [18]; risk factors for skeletal fragility (e.g., previous fragility fractures, family history of fractures, smoking habits, and glucocorticoid use), along with calculation of the FRAX score (not considering index HF and not including bone mineral density data) [19]; data on prior vitamin D supplementation and anti-fracture treatment. A 12-month follow-up was planned either by telephone contact with patients or their relatives, or through in-person visits, to assess clinical outcomes including refracture and mortality.

### Laboratory tests

Laboratory tests were conducted at the Laboratory Medicine Unit of the University Hospital of Padua, utilizing methods monitored for quality performance in accordance with the ISO 15,189 standard. Laboratory tests were performed within 24–48 h after hospital admission, before orthopedic surgery. Lithium-heparin plasma samples were collected for calcium and phosphate measurements using colorimetric methods, and for creatinine measurement via an enzymatic assay (calibrated to the reference procedure). The estimated glomerular filtration rate (eGFR) was calculated according to the CKD-EPI (Chronic Kidney Disease Epidemiology Collaboration) formula [20]. Serum 25(OH)D and PTH (third-generation assay, reference range 6.5–36.8 ng/L) levels were measured using automated immunochemiluminescent methods (Liaison XL, DiaSorin, Saluggia, Italy). VDD was defined as serum 25(OH)D levels below 50 nmol/L [8, 9]. Alkaline phosphatase (ALP) activity was measured in lithium-heparin plasma using an enzymatic-colorimetric method (Roche Diagnostics, Penzberg, Germany). Bone-specific alkaline phosphatase (BALP), C-terminal telopeptide of type I collagen (CTX), and procollagen type 1 N-terminal propeptide (P1NP) were assayed using an immunochemiluminescent method on an IDS-iSYS system (Pantec, Torino, Italy).

### Statistical analysis

Categorical variables are presented as frequencies and percentages, while continuous variables are expressed as mean ± standard deviation or median (interquartile range), as appropriate. The normal distribution of continuous variables was assessed using the Shapiro-Wilk test. For the entire cohort (*n* = 934), differences between patients with and without VDD were assessed using the Student’s t-test or the Mann–Whitney U test for continuous variables, and the chi-squared test or Fisher’s exact test for categorical variables. Correlation analysis between laboratory parameters was performed using Spearman correlation coefficients. Multivariable logistic regression models were used to identify patient characteristics associated with the presence of VDD and with the use of vitamin D supplements prior to hospital admission. Variables with a p-value < 0.10 in univariate analysis were considered for inclusion in the multivariable models. Clinically relevant variables were retained regardless of their univariate p-value. Collinearity among predictors was assessed using the Variance Inflation Factor (VIF), and variables with VIF > 5 were excluded. Results are reported as odds ratios (OR) with 95% confidence intervals (95% CI). To develop a practical clinical tool for VDD risk stratification, a classification and regression tree (CART) analysis was performed to identify patient phenotypes at highest risk of vitamin D deficiency using variables identified as significant predictors in the multivariable logistic regression. The decision tree was built with a maximum depth of 3 levels to ensure interpretability, requiring a minimum of 50 patients per leaf and 100 patients per split. The dataset was randomly divided into training (70%) and testing (30%) sets. Model performance was assessed using the area under the receiver operating characteristic curve (AUC) with 95% CI. To optimize model parsimony, backward elimination was applied based on feature importance, removing variables with minimal predictive contribution. The final model identified distinct patient phenotypes with corresponding VDD prevalence rates. In the subcohort of patients for whom 12-month follow-up data were available (*n* = 554), clinical outcomes at 12 months were assessed after adjustment for baseline differences using inverse probability of treatment weighting (IPTW) based on age, sex, and CCI [21]. The relationship between VDD and clinical outcomes at 12 months after HF was analyzed using Cox proportional hazards regression models, with results expressed as hazard ratios (HR) and 95% CI. Additionally, survival analysis was performed using Kaplan–Meier curves, and differences in survival between patients with and without VDD were assessed using the log-rank test. To complement the categorical VDD analysis, we performed Cox proportional hazards regression treating 25(OH)D as a continuous variable. HR were calculated relative to a reference level of 50 nmol/L. Statistical analysis was performed using Python, with the significance level set at *p* < 0.05.

## Results

### Clinical and laboratory characteristics of the patients

**Fig. 1:**
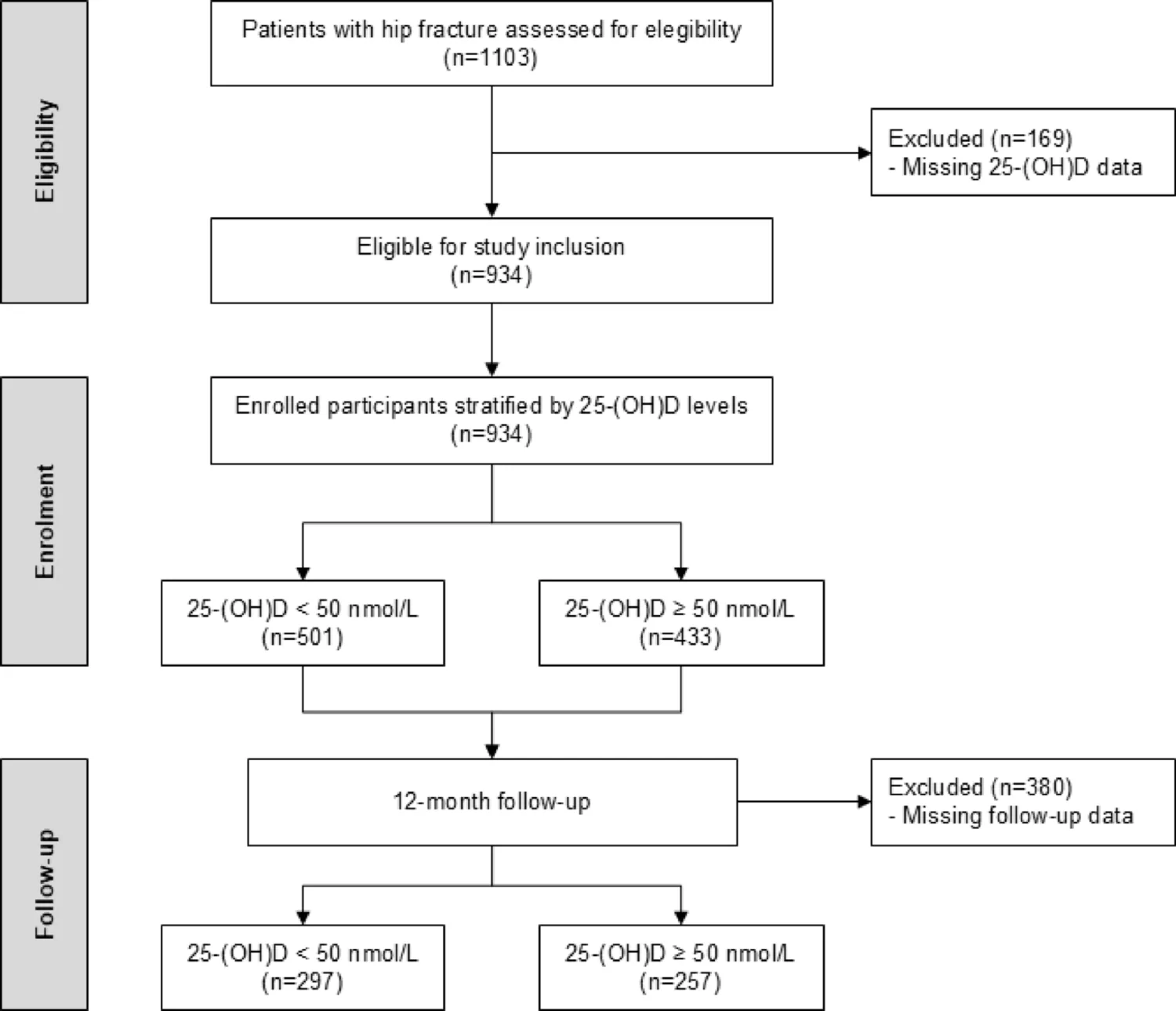
Study flow diagram. STROBE flow diagram showing participant enrollment, stratification by 25(OH)D levels, and 12-month follow-up in a prospective cohort study of hip fracture patients

Between March 2023 and March 2025, a total of 1103 patients were assessed within our FLS program. Of these, 169 were excluded from this study due to missing 25(OH)D data, as shown in Fig. 1. A total of 934 patients were included, with a mean age of 83.0 ± 8.9 years; 71.2% were female. Among them, 501 (53.6%) had VDD, while 433 (46.4%) did not. Clinical characteristics of participants at admission, stratified by serum 25(OH)D levels, are summarized in Table 1. Subjects with VDD were slightly but not significantly older than those with 25(OH)D ≥ 50 nmol/L (*p* = 0.084), and women were more prevalent in the higher 25(OH)D group (*p* < 0.001). Participants with VDD had a higher BMI (*p* < 0.001) and a greater number of comorbidities, including previous myocardial infarction (*p* = 0.020), heart failure (*p* = 0.049), diabetes mellitus (*p* < 0.001), chronic obstructive pulmonary disease (COPD) (*p* = 0.007), chronic kidney disease (CKD) stage 4–5 (*p* = 0.003), and dementia (*p* < 0.001). The cumulative comorbidity burden, assessed with the CCI, was significantly higher in patients with VDD (5.5 ± 2.1 vs. 5.1 ± 1.9, *p* < 0.001). Regarding risk factors for fractures, a higher proportion of subjects with 25(OH)D ≥ 50 nmol/L at hospital admission reported a history of previous fractures (*p* = 0.014), including major osteoporotic fractures (MOF) (28% vs. 19%, *p* = 0.002), and clinical vertebral fractures (14% vs. 8%, *p* = 0.006). Furthermore, prior use of vitamin D supplements (60% vs. 15%, *p* < 0.001) and anti-fracture treatments (14% vs. 4%, *p* < 0.001) were significantly more frequent in the 25(OH)D ≥ 50 nmol/L group.

**Table 1:** Clinical characteristics of patients at admission

	25-(OH)D < 50 nmol/L (*n* = 501)	25-(OH)D ≥ 50 nmol/L (*n* = 433)	*p*
Age (year), mean (SD)	83.4 (9.1)	82.4 (8.7)	0.084
Female sex, n (%)	303 (60)	362 (84)	**< 0.001**
BMI (kg/m^2^), mean (SD)	24.6 (4.7)	23.6 (4.0)	**< 0.001**
*Comorbidities*			
Hypertension, n (%)	347 (69)	283 (65)	0.208
Previous myocardial infarction, n (%)	72 (14)	40 (9)	**0.020**
Heart failure, n (%)	66 (13)	39 (9)	**0.049**
Previous venous thromboembolism, n (%)	28 (6)	23 (5)	0.886
Previous stroke, n (%)	55 (11)	35 (8)	0.149
Diabetes mellitus, n (%)	113 (23)	59 (14)	**< 0.001**
COPD, n (%)	46 (9)	20 (5)	**0.007**
Rheumatic diseases, n (%)	15 (3)	36 (8)	**< 0.001**
Liver cirrhosis, n (%)	6 (1)	4 (1)	0.759
CKD stage 4–5, n (%)	28 (6)	8 (2)	**0.003**
Dementia, n (%)	146 (29)	76 (18)	**< 0.001**
CCI, mean (SD)	5.5 (2.1)	5.1 (1.9)	**0.002**
*Risk factors and treatment for skeletal fragility*			
Family history of osteoporosis, n (%)	81 (16)	94 (22)	**0.035**
Active smoking, n (%)	50 (10)	24 (6)	**0.015**
Alcohol abuse, n (%)	14 (3)	4 (1)	0.054
Glucocorticoid use, n (%)	10 (2)	14 (3)	0.300
At least one previous fracture, n (%)	146 (29)	159 (37)	**0.014**
At least one previous MOF, n (%)	97 (19)	122 (28)	**0.002**
At least one previous HF, n (%)	23 (5)	32 (7)	0.137
At least one previous vertebral fracture, n (%)	40 (8)	59 (14)	**0.006**
FRAX MOF (%)*, median (IQR)	17.0 (9.3–24.0)	22.0 (14.0–31.0)	**< 0.001**
Previous vitamin D supplements, n (%)	73 (15)	260 (60)	**< 0.001**
Previous anti-fracture treatment, n (%)	19 (4)	59 (14)	**< 0.001**

25-(OH)D: 25-(OH) vitamin D, BMI: body mass index, CCI: Charlson Comorbidity Index, CKD: chronic kidney disease, COPD: chronic obstructive pulmonary disease, FRAX: fracture risk assessment tool, HF: hip fracture, IQR: interquartile range, MOF: major osteoporotic fracture, SD: standard deviation. *Calculated not including index hip fracture. Significant p-values are highlighted in bold

Laboratory tests at admission are shown in Table 2. Patients with VDD had significantly lower total calcium (*p* = 0.009) and phosphate levels (*p* = 0.004) compared with those with sufficient vitamin D levels. As expected, 25(OH)D levels were lower in the VDD group (22.0 [14.0–35.0] vs. 73.0 [59.0–88.0] nmol/L, *p* < 0.001), whereas PTH levels were higher (*p* < 0.001). ALP, BALP, and CTX were also significantly higher in VDD patients (*p* < 0.001 for all comparisons). We also found slight differences in creatinine and eGFR (*p* < 0.001 and *p* = 0.004, respectively). To further explore the relationships between laboratory parameters, we performed correlation analysis treating serum 25(OH)D as a continuous variable. As shown in **Figure ****S1**, correlation analysis confirmed a moderate inverse association between 25(OH)D levels and PTH (ρ=-0.336, *p* < 0.001), and weak but significant negative correlations with bone turnover markers including BALP (ρ=-0.169, *p* < 0.001), CTX (ρ=-0.159, *p* < 0.001), and ALP (ρ=-0.127, *p* < 0.001). Additionally, 25(OH)D showed weak positive correlations with calcium (ρ = 0.132, *p* < 0.001) and phosphate (ρ = 0.120, *p* < 0.001) levels, as well as negative and positive correlations with creatinine (ρ=-0.152, *p* < 0.001) and eGFR (ρ = 0.093, *p* < 0.01), respectively.

**Table 2:** Laboratory tests at admission in patients with and without VDD

	25-(OH)D < 50 nmol/L (*n* = 501)	25-(OH)D ≥ 50 nmol/L (*n* = 433)	*p*
Creatinine (mg/dL)	0.80 (0.66–1.05)	0.72 (0.60–0.92)	< 0.001
eGFR (mL/min/1.73 m²)	80.0 (57.0–89.0)	84.0 (65.0–92.0)	**0.004**
Calcium (mg/dL)	8.60 (8.28–8.88)	8.68 (8.32-9.00)	**0.009**
Phosphate (mg/dL)	2.76 (2.38–3.28)	2.94 (2.48–3.44)	**0.004**
25-(OH) vitamin D (nmol/L)	22.0 (14.0–35.0)	73.0 (59.0–88.0)	**< 0.001**
PTH (ng/L)	39.4 (28.9–54.0)	31.5 (23.6–41.2)	**< 0.001**
ALP (U/L)	73.5 (59.0–93.0)	68.0 (55.0–86.0)	**< 0.001**
BALP (µg/L)	14.5 (11.2–18.6)	12.7 (9.62–16.6)	**< 0.001**
CTX (pg/ml)	455 (282–681)	349 (220–586)	**< 0.001**
P1NP (µg/L)	41.5 (30.1–59.3)	38.5 (27.3–56.9)	0.059

Data are presented as median and interquartile range. Abbreviations: 25-(OH)D: 25-(OH) vitamin D, ALP: alkaline phosphatase, BALP: bone-specific alkaline phosphatase, CTX: C-terminal telopeptide of type I collagen, eGFR: estimated glomerular filtration rate, P1NP: procollagen type 1 N-terminal propeptide, PTH: parathyroid hormone. Significant p-values are highlighted in bold

### Factors associated with VDD and vitamin D supplementation

**Fig. 2:**
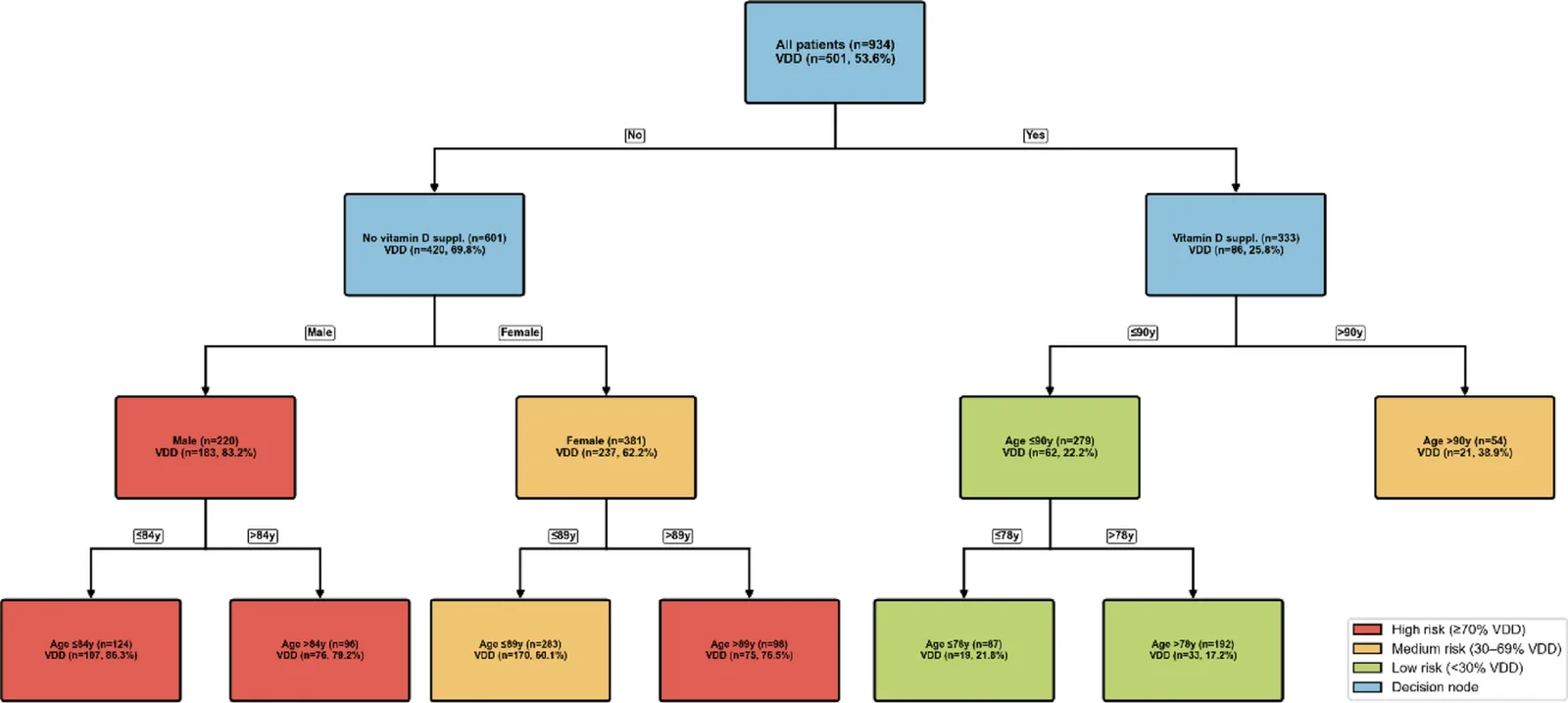
Decision tree for vitamin D deficiency risk stratification in hip fracture patients. Classification tree showing seven patient phenotypes based on vitamin D supplementation, sex, and age. Nodes are color-coded by vitamin D deficiency (VDD) risk: red (≥ 70%), orange (30–69%), green (< 30%), blue (decision nodes)

Following the characterization of the population and its laboratory profiles, we subsequently conducted a logistic regression analysis to assess which factors were associated with VDD, as shown in Table 3. In the multivariable model, male sex (*p* < 0.001), diabetes mellitus (*p* = 0.003), dementia (*p* = 0.013), and active smoking (*p* = 0.045) were identified as independent predictors of VDD, whereas vitamin D supplementation prior to hospitalization was inversely associated (*p* < 0.001). To further enhance clinical interpretability and develop a practical tool for identifying patients at highest risk of VDD, we constructed a decision tree analysis incorporating the variables identified in the multivariable logistic regression. We included male sex, diabetes mellitus, dementia, active smoking, and previous vitamin D supplementation, along with age (which approached statistical significance in logistic regression analysis, *p* = 0.050), given its potential clinical importance. The initial decision tree model using all six variables achieved an AUC of 0.788 and revealed that previous vitamin D supplementation dominated the predictive power (feature importance: 86.7%), followed by sex (9.4%) and age (3.9%), while diabetes, dementia, and smoking contributed minimally to the classification. To optimize model parsimony without compromising predictive performance, we applied backward elimination based on feature importance analysis. Variables with limited contribution to the decision tree’s discriminative ability were systematically removed. The final decision tree model retained three variables: previous vitamin D supplementation, sex, and age. This simplified model maintained equivalent predictive performance (AUC 0.788, 95% CI 0.734–0.840, *p* < 0.001). As shown in Fig. 2, this model identified distinct patient phenotypes with VDD prevalence ranging from 17.2% to 86.3%. The decision tree revealed that patients without vitamin D supplementation had consistently high VDD rates (60.1% to 86.3%), with men showing the highest risk regardless of age, while all patients with supplementation demonstrated substantially lower VDD prevalence (17.2% to 38.9%), highlighting the dominant protective effect of vitamin D supplementation in HF patients.

**Table 3:** Logistic regression analysis for factors associated with VDD

	Univariable analysis		Multivariable analysis	
Predictor Variable	OR (95% CI)	*p*	OR (95% CI)	*p*
Age (10 years)	1.14 (0.98–1.31)	0.086	1.21 (1.00-1.47)	0.050
Male sex	3.33 (2.44–4.55)	**< 0.001**	2.21 (1.50–3.24)	**< 0.001**
BMI (kg/m^2^)	1.05 (1.02–1.09)	**0.001**	1.03 (1.00-1.07)	0.077
*Comorbidities*				
Hypertension	1.19 (0.91–1.57)	0.204	-	-
Previous myocardial infarction	1.65 (1.09–2.48)	**0.017**	0.92 (0.55–1.53)	0.739
Heart failure	1.53 (1.01–2.33)	**0.046**	1.05 (0.62–1.77)	0.861
Previous venous thromboembolism	1.06 (0.60–1.86)	0.853	-	-
Previous stroke	1.40 (0.90–2.19)	0.136	-	-
Diabetes mellitus	1.85 (1.31–2.61)	**< 0.001**	1.91 (1.24–2.93)	**0.003**
COPD	2.09 (1.21–3.59)	**0.008**	1.21 (0.63–2.34)	0.566
Rheumatic diseases	0.34 (0.18–0.63)	**< 0.001**	0.55 (0.26–1.16)	0.116
Liver cirrhosis	1.30 (0.36–4.64)	0.686	-	-
CKD stage 4–5	3.14 (1.42–6.98)	**0.005**	2.46 (0.90–6.69)	0.079
Dementia	1.93 (1.41–2.64)	**< 0.001**	1.63 (1.11–2.40)	**0.013**
CCI	1.11 (1.04–1.19)	**0.002**	-	-
*Risk factors and treatment for skeletal fragility*				
Family history of osteoporosis	0.70 (0.50–0.97)	**0.031**	0.98 (0.64–1.49)	0.915
Active smoking	1.89 (1.14–3.13)	**0.013**	1.96 (1.02–3.80)	**0.045**
Alcohol abuse	3.08 (1.01–9.44)	**0.049**	1.30 (0.35–4.88)	0.695
Glucocorticoid use	0.61 (0.27–1.39)	0.238	-	-
At least one previous fracture	0.71 (0.54–0.93)	**0.014**	1.05 (0.75–1.49)	0.765
At least one previous MOF	0.61 (0.45–0.83)	**0.002**	-	-
At least one previous HF	0.75 (0.46–1.21)	0.242	-	-
At least one previous vertebral fracture	0.55 (0.36–0.84)	**0.006**	-	-
FRAX MOF (%)*, median (IQR)	0.96 (0.95–0.98)	**< 0.001**	-	-
Previous vitamin D supplements	0.11 (0.08–0.16)	**< 0.001**	0.14 (0.10–0.19)	**< 0.001**

95% CI: 95% confidence interval, BMI: body mass index, CCI: Charlson Comorbidity Index, CKD: chronic kidney disease, COPD: chronic obstructive pulmonary disease, FRAX: fracture risk assessment tool, HF: hip fracture, MOF: major osteoporotic fracture, OR: odds ratio, VDD: Vitamin D deficiency. *Calculated not including index hip fracture. Significant p-values are highlighted in bold

Given that vitamin D supplementation emerged as the dominant predictor in our decision tree model, we decided to perform separate logistic regression analyses to identify factors associated with VDD in distinct patient subgroups. First, we analyzed patients who were not taking vitamin D supplementation before hospitalization (*n* = 601, 64.3%). As shown in Table S1, age (*p* = 0.014), male sex (*p* < 0.001), diabetes mellitus (*p* = 0.017) and active smoking (*p* = 0.015) were independently associated with VDD in the multivariable analysis in this cohort. Subsequently, we analyzed patients who were taking vitamin D at home before hospitalization (*n* = 333, 35.7%). As reported in **Table S2**, COPD (*p* = 0.045) and dementia (*p* = 0.012) were independent predictors of VDD in this cohort.

Finally, given that different risk factors emerged in supplemented versus non-supplemented patients, we investigated which baseline characteristics influenced the likelihood of receiving home vitamin D supplementation before the index fracture. As shown in Table S3, female sex, rheumatic disease, family history of osteoporosis, and a history of previous fractures were associated with higher odds of home vitamin D supplementation (*p* < 0.001, *p* = 0.004, *p* = 0.015, and *p* = 0.001, respectively). Conversely, dementia (*p* = 0.003) was associated with lower odds of supplementation.

### In-hospital complications and 12-month outcomes

**Fig. 3:**
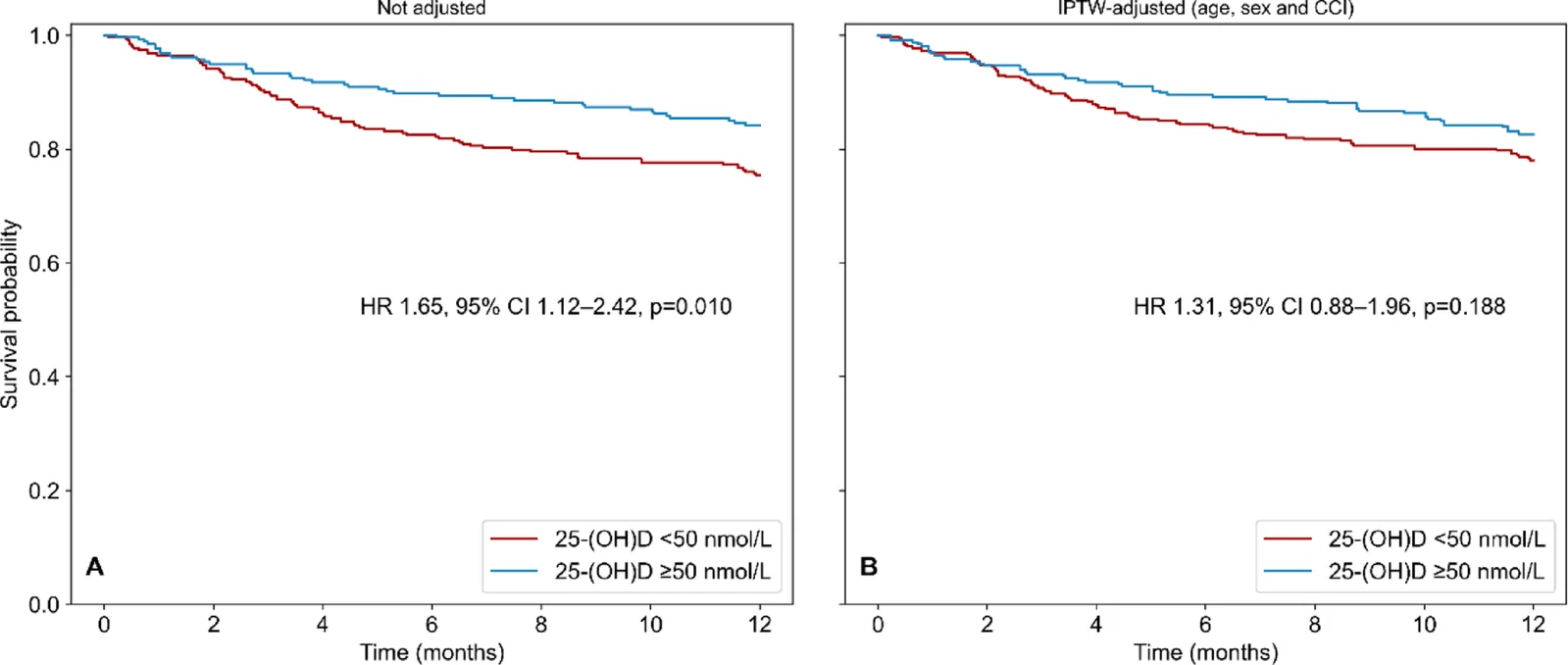
12-month survival by baseline 25(OH)D levels. (**A**) Unadjusted Kaplan–Meier curves. (**B**) IPTW-weighted curves (adjusted for age, sex, and CCI). Abbreviations: 25(OH)D: 25-(OH) vitamin D, 95% CI: 95% confidence interval, CCI: Charlson Comorbidity Index, HR: Hazard ratio, IPTW: Inverse probability of treatment weighting

As shown in Figs. 1 and 12-month follow-up data were available for 554 of the 934 included patients. However, the demographic and clinical characteristics of this subgroup were largely comparable to those of patients without available follow-up data, except for a higher prevalence of previous fractures among patients with available follow-up data, as shown in Table S4. Clinical outcomes during hospitalization among the 554 patients with available follow-up data are summarized in Table 4. No differences were observed in major in-hospital complications or length of hospital stay between patients with and without VDD (*p* = 0.348). Regarding long-term outcomes, the events observed up to 12 months after the index fracture in patients with and without VDD are also shown in Table 4. Refracture and rehospitalization rates did not differ between the groups, whereas the number of deaths at 12 months was higher in patients with VDD (25% vs. 16%; *p* = 0.013). To explore this outcome while accounting for differences in comorbidity burden, we applied IPTW based on age, sex, and CCI as key mortality confounders, generating an IPTW pseudo-population of 1124 patients. Kaplan–Meier survival analysis was then performed to compare 12-month mortality between the groups. In the unadjusted analysis (Fig. 3A), VDD was associated with a significantly higher hazard of death (HR 1.65, 95% CI 1.12–2.42; *p* = 0.010). After IPTW adjustment, the association was attenuated and no longer statistically significant (HR 1.31, 95% CI 0.88–1.96; *p* = 0.188), as shown in Fig. 3B. We also performed IPTW-adjusted analyses separately in the subgroup of patients who were not receiving home vitamin D supplementation and in those who were treated prior to the index fracture. In neither subgroup was VDD associated with a higher mortality rate (Figure S2).

**Table 4:** In-hospital complications and 12-month outcomes in patients with and without VDD

	25-(OH)D < 50 nmol/L (*n* = 297)	25-(OH)D ≥ 50 nmol/L (*n* = 257)	*p*
In-hospital complications			
Acute heart failure episode, n (%)	24 (8)	23 (9)	0.831
Acute myocardial injury, n (%)	21 (7)	13 (5)	0.420
Tachyarrhythmia, n (%)	15 (5)	18 (7)	0.430
Infection, n (%)	65 (22)	69 (27)	0.207
COVID-19, n (%)	27 (9)	18 (7)	0.459
Acute kidney injury, n (%)	15 (5)	13 (5)	1.000
Venous thromboembolism, n (%)	9 (3)	10 (4)	0.748
Stroke/TIA, n (%)	3 (1)	5 (2)	0.573
ICU transfer, n (%)	9 (3)	8 (3)	1.000
Length of stay (days), median (IQR)	12.0 (9.0-16.5)	12.5 (10.0–16.0)	0.348
*Post-discharge outcomes*			
1-month death, n (%)	12 (4)	5 (2)	0.238
12-months death, n (%)	74 (25)	41 (16)	**0.013**
12-months new fracture, n (%)	12 (4)	15 (6)	0.435
12-months new hospitalization, n (%)	62 (21)	54 (21)	1.000

25-(OH)D: 25-(OH) vitamin D, COVID-19: coronavirus disease 2019, ICU: intensive care unit, IQR: interquartile range, TIA: transient ischemic attack, VDD: vitamin D deficiency. Significant p-values are highlighted in bold

**Fig. 4:**
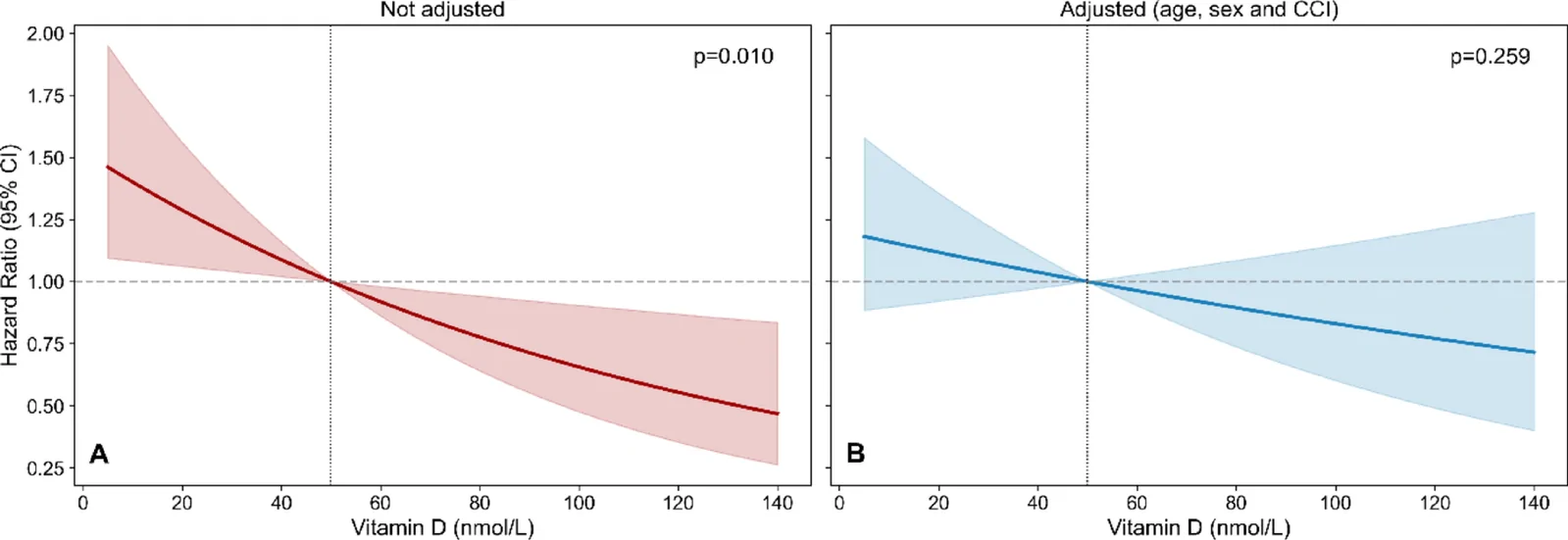
Association between baseline 25(OH)D levels and 12-month mortality: (**A**) unadjusted and (**B**) adjusted hazard ratios with 95% CI. Abbreviations: 25(OH)D: 25-(OH) vitamin D, 95% CI: 95% confidence interval, CCI: Charlson Comorbidity Index. Reference level: 50 nmol/L

To complement the dichotomous VDD analysis, we further explored the association between serum vitamin D levels and 12-month mortality by modeling 25(OH)D as a continuous variable using Cox proportional hazards regression. Hazard ratios were calculated and plotted relative to a reference value of 50 nmol/L. In unadjusted analysis (Fig. 4A), each nmol/L increase in vitamin D reduced mortality risk by 0.8% (HR 0.992, *p* = 0.010), with severe deficiency (25 nmol/L) associated with 22% higher mortality risk versus the 50 nmol/L reference. However, in concordance with the dichotomous VDD analysis, after adjusting for age, sex, and comorbidity burden (Fig. 4B), this association became non-significant (HR 0.996, *p* = 0.259), with effect sizes substantially attenuated across all vitamin D levels.

Finally, analysis of secondary outcomes revealed no significant associations between VDD and either 12-month refracture or rehospitalization rates in both unadjusted and IPTW-adjusted models (*p* > 0.05 for all comparisons).

## Discussion

The present study investigated the prevalence and clinical correlates of VDD in real-world practice at a large tertiary referral center within an established FLS program [16]. This program, initiated in 2023, aims to optimize osteoporosis management and prevent secondary fractures in patients admitted with HF. As part of the comprehensive bone health assessment, serum 25(OH)D levels were systematically measured at admission. In our cohort, VDD was present in 53.6% of patients, consistent with the 34–80% prevalence reported in other HF populations using the 25(OH)D < 50 nmol/L definition [22]. This high prevalence underscores VDD as a common clinical concern in HF patients.

Patients with VDD in our cohort displayed significantly worse clinical characteristics, including higher BMI and a greater burden of both individual comorbidities—such as diabetes mellitus, dementia, chronic kidney disease, and cardiovascular disease—and overall comorbidity burden, as measured by the CCI. While these associations align with previous literature [23–28], the causal relationship remains uncertain. Many comorbid conditions are linked to reduced vitamin D status through mechanisms such as decreased mobility and sun exposure, gastrointestinal malabsorption, chronic inflammation, or impaired hepatic and renal metabolism [29]. This appears to be the case in our study, where VDD is more likely to represent a marker of underlying clinical vulnerability rather than an independent driver of adverse outcomes [30]. However, these findings are not unexpected given the very advanced age of our patients and the substantial comorbidity burden characterizing this population, both of which heavily condition overall life expectancy [31].

Interestingly, patients with adequate vitamin D levels (≥ 50 nmol/L) exhibited a higher prevalence of prior fractures and higher FRAX scores compared with those with VDD, and were more likely to have been receiving both vitamin D supplementation and anti-fracture therapy before the index fracture. Although paradoxical at first glance, this observation could reflect that patients at higher fracture risk had, at least in part, already been identified, evaluated for skeletal fragility and managed accordingly.

Beyond describing baseline characteristics, we explored predictors of VDD using multivariable logistic regression. Male sex, diabetes mellitus, dementia, and active smoking emerged as independent risk factors, whereas prior vitamin D supplementation was strongly protective. To better understand these relationships, decision tree analysis revealed supplementation as the most influential factor (feature importance 86.7%), with sex (9.4%) and age (3.9%) playing secondary roles. The final simplified model identified patient phenotypes with VDD prevalence ranging from 17.2% to 86.3%. At the low-risk end, younger females on supplementation had the lowest prevalence, whereas at the high-risk end, older males without supplementation showed rates exceeding 86%. Notably, this high-risk group also appears to be undertested, as previous studies have documented fewer vitamin D tests administered to the oldest male cohorts compared to other demographic groups [32]. Such stratification may assist clinicians in recognizing patients at very high risk of severe VDD who might require empirical supplementation to achieve and maintain adequate vitamin D levels [33].

Nevertheless, the lack of vitamin D supplementation before admission was very frequent. Although vitamin D supplementation is recommended alongside anti-fracture treatment for subjects with increased fracture risk [4, 33], and the Endocrine Society suggests empirical vitamin D treatment in certain high-risk populations (including individuals aged 75 years and older and those with high-risk prediabetes) [8], the percentage of patients receiving such therapy at home in our cohort was low (35.7%), though consistent with previously reported rates in the literature [34]. Given all these data, our study underscores that supplementing vitamin D constitutes the only way to avoid VDD in this cohort of old, frail and comorbid patients.

In addition, considering the supplementation’s dominant role in risk prediction, we conducted subgroup analyses by vitamin D treatment status. In non-supplemented patients, classical risk factors (age, male sex, diabetes, smoking) predominated. In supplemented patients, COPD and dementia emerged as main predictors of persistent deficiency, suggesting standard supplementation may be insufficient in these vulnerable groups. COPD patients may exhibit increased catabolism due to chronic systemic inflammation [35], while patients with cognitive impairment demonstrate poor medication adherence [36].

We then examined clinical outcomes, with 12-month mortality as the primary endpoint. In crude analysis of our cohort, VDD was associated with significantly higher mortality rates, and both dichotomous Kaplan-Meier analysis and continuous Cox regression demonstrated significant associations with 12-month mortality. However, we sought to isolate the independent contribution of vitamin D levels from other factors associated with mortality, such as age, sex, and comorbidity burden. After appropriate adjustment for these confounding variables, the associations between VDD and mortality were attenuated and lost statistical significance. From our study it emerges that, when evaluating the association between VDD and mortality, comorbidity burden and overall health status should always be taken into account as the major determinants of this relationship, especially when considering advanced age and very frail populations [30, 37, 38].

Rehospitalization rates did not differ between groups, which is clinically plausible given that rehospitalization in HF patients is typically driven by acute medical events and surgical complications rather than vitamin D status. Similarly, refracture rates at 12 months were comparable between groups. Although this may appear unexpected given the well-established biological role of vitamin D in bone metabolism, the relatively short follow-up period likely limited the number of fracture events observed. Indeed, previous studies within FLS models have shown that at least 15 months of follow-up are typically required for fracture events to become sufficiently frequent to demonstrate meaningful clinical trends [39].

Beyond clinical outcomes, the biochemical profile of patients with VDD suggests a plausible pathophysiological mechanism underlying their increased skeletal fragility. The elevations in PTH, BALP, and CTX indicate secondary hyperparathyroidism and accelerated bone turnover. These alterations are largely attributable to VDD itself, reflecting an adaptive response to impaired calcium absorption and disrupted mineral homeostasis. Such metabolic changes could be indicative of compromised bone health and may represent key pathophysiological drivers of skeletal fragility in this population [40]. From a clinical perspective, our findings should not be interpreted as evidence supporting routine 25(OH)D testing, as this was beyond the scope of the present study. Rather, they may help identify subgroups of HF patients at particularly high risk of VDD who could benefit from prompt optimization of vitamin D status, as correction of VDD is often required before or alongside rapid initiation of anti-fracture treatment. Although no universal consensus currently exists regarding the optimal replacement strategy in the acute HF setting, Italian national guidelines suggest cholecalciferol doses ranging from 3000 to 10,000 IU/day (or equivalent weekly regimens) for 1–2 months in patients with VDD [33]. However, such timeframes may be impractical in the acute phase after HF, when timely initiation of anti-osteoporotic therapy is desirable because of the highly imminent refracture risk. Alternative loading strategies have therefore been proposed. In particular, a single oral dose of 250,000 IU vitamin D has been shown to be safe and effective after HF, with improvement 25(OH)D levels already observed after two weeks [41], while some authors have recently suggested loading regimens of 150,000–250,000 IU administered in divided doses over 1–7 days, preferably during the index hospitalization [42].

Strengths of our study include the large sample size, systematic assessment of vitamin D in a real-world HF population, and the use of multiple complementary methods to address confounding. Several limitations should be also acknowledged. Home vitamin D supplementation was assessed through patient self-report and medical records, without confirmation of dosing regimens or strict treatment adherence. Nevertheless, supplementation history consistently emerged as the strongest predictor of serum 25(OH)D in our cohort, supporting the reliability of the reported supplementation status and suggesting that measured vitamin D levels likely reflected previous vitamin D exposure in many patients. In addition, serum 25(OH)D was measured during the acute post-fracture phase, when inflammation-related reductions in circulating vitamin D may occur [43]. However, blood sampling was performed early after admission and before orthopedic surgery, theoretically limiting acute-phase related fluctuations, and a relatively conservative threshold was adopted for defining VDD (< 50 nmol/L), whereas higher target levels have been proposed for patients with skeletal fragility [33]. Therefore, despite the potential influence of the acute-phase response, the overall burden of inadequate vitamin D status in this population was unlikely to have been substantially overestimated. We also relied on single time-point measurements of 25(OH)D, which may not capture long-term status or seasonal variation, although continuous enrolment over two years likely reduced seasonal bias. Finally, the 12-month follow-up, while relevant for short-term outcomes, may not have been sufficient to detect longer-term effects of VDD on fracture risk, which often require extended observation to become clinically apparent.

In conclusion, VDD is highly prevalent among HF patients, particularly in older males not receiving supplements. While VDD was not independently associated with mortality, it appears to reflect underlying comorbidity status. VDD may be associated with worse bone health compared to HF patients without VDD, but longer follow-up periods are needed to establish clear associations with fracture risk. The very high prevalence of VDD observed in this population suggests that empirical vitamin D supplementation may represent a reasonable strategy in many patients, while identification of specific high-risk phenotypes may help guide individualized assessment and management.

## Supplementary Information

Below is the link to the electronic supplementary material.

Supplementary Material 1

## Data Availability

The datasets analyzed during the current study are available from the corresponding author on reasonable request.
