# Supplementary material for: Prevalence, predictors, and clinical impact of vitamin D deficiency on 12-month outcomes in hip fracture patients
Source: J Endocrinol Invest. 2026 Jun 27;49(10):2913–24. doi: 10.1007/s40618-026-02952-x (PMC13627231; doi:10.1007/s40618-026-02952-x)
Supplement: Supplementary file 1 [file 40618_2026_2952_MOESM1_ESM.pdf]

Prevalence, Predictors, and Clinical Impact of Vitamin D Deficiency on 12-Month Outcomes in Hip Fracture Patients

Supplementary data

Table of contents

Table S1. Logistic regression analysis for factors associated with VDD in patients not taking home vitamin D supplements...2

Table S2. Logistic regression analysis for factors associated with VDD in patients taking home vitamin D supplements...3

Table S3. Logistic regression analysis for factors associated with home vitamin D supplementation. 4

Table S4. Baseline characteristics of patients with and without 12-month follow-up data ...5

Figure S1. Correlation matrix of laboratory tests. 6

Figure S2. 12-month survival by baseline 25-(OH)D levels in patients without (A) and with prior vitamin D supplementation (B). 7

**Table S1. Logistic regression analysis for factors associated with VDD in patients not taking home vitamin D supplements.**				
	**Univariable analysis**		**Multivariable analysis**	
**Predictor Variable**	**OR (95% CI)**	**p**	**OR (95% CI)**	**p**
Age (10 years)	1.13 (0.93-1.36)	0.213	1.32 (1.06-1.65)	**0.014**
Male sex	2.75 (1.82-4.14)	**<0.001**	2.59 (1.67-4.01)	**<0.001**
BMI (kg/m^2)	1.05 (1.00-1.09)	**0.032**	1.04 (0.99-1.08)	0.086
Comorbidities				
Hypertension	1.22 (0.84-1.76)	0.300	-	-
Previous myocardial infarction	1.40 (0.81-2.42)	0.229	-	-
Heart failure	1.20 (0.70-2.06)	0.511	-	-
Previous venous thromboembolism	1.17 (0.51-2.67)	0.707	-	-
Previous stroke	1.59 (0.84-3.01)	0.154	-	-
Diabetes mellitus	2.18 (1.30-3.67)	**0.003**	1.96 (1.13-3.42)	**0.017**
COPD	1.48 (0.74-2.95)	0.271	-	-
Rheumatic diseases	0.54 (0.22-1.38)	0.199	-	-
Liver cirrhosis	1.21 (0.13-11.75)	0.867	-	-
CKD stage 4-5	3.52 (1.05-11.80)	**0.042**	2.46 (0.72-8.48)	0.153
Dementia	1.48 (0.97-2.25)	0.066	1.40 (0.88-2.22)	0.150
CCI	1.11 (1.01-1.22)	**0.026**	-	-
Risk factors and treatment for skeletal fragility				
Family history of osteoporosis	0.76 (0.48-1.21)	0.251	-	-
Active smoking	1.92 (0.94-3.89)	0.073	2.86 (1.22-6.70)	**0.015**
Alcohol abuse	2.68 (0.60-12.00)	0.198	-	-
Glucocorticoid use	0.40 (0.11-1.39)	0.148	-	-
At least one previous fracture	0.93 (0.63-1.37)	0.698	-	-
At least one previous MOF	0.79 (0.51-1.22)	0.293	-	-
At least one previous HF	0.77 (0.38-1.56)	0.473	-	-
At least one previous vertebral fracture	0.79 (0.43-1.46)	0.454	-	-
FRAX MOF (%)*, median (IQR)	0.97 (0.96-0.99)	**0.003**	-	-

Abbreviations: 95% CI: 95% confidence interval, BMI: body mass index, CCI: Charlson Comorbidity Index, CKD: chronic kidney disease, COPD: chronic obstructive pulmonary disease, FRAX: fracture risk assessment tool, HF: hip fracture, MOF: major osteoporotic fracture, OR: odds ratio, VDD: Vitamin D deficiency. *Calculated not including index hip fracture. Significant p-values are highlighted in bold.

**Table S2. Logistic regression analysis for factors associated with VDD in patients taking home vitamin D supplements.**				
	**Univariable analysis**		**Multivariable analysis**	
**Predictor Variable**	**OR (95% CI)**	**p**	**OR (95% CI)**	**p**
Age (10 years)	1.22 (0.88-1.70)	0.224	1.06 (0.75-1.51)	0.726
Male sex	1.72 (0.88-3.37)	0.114	1.45 (0.71-2.97)	0.306
BMI (kg/m^2)	1.03 (0.97-1.10)	0.343	-	-
Comorbidities				
Hypertension	1.66 (0.91-3.02)	0.100	-	-
Previous myocardial infarction	1.60 (0.70-3.66)	0.266	-	-
Heart failure	1.56 (0.65-3.73)	0.316	-	-
Previous venous thromboembolism	1.20 (0.42-3.42)	0.732	-	-
Previous stroke	0.97 (0.38-2.49)	0.947	-	-
Diabetes mellitus	1.85 (0.99-3.49)	0.055	1.83 (0.95-3.53)	0.069
COPD	2.96 (1.06-8.24)	**0.038**	3.15 (1.03-9.67)	**0.045**
Rheumatic diseases	0.48 (0.16-1.42)	0.184	-	-
Liver cirrhosis	3.67 (0.73-18.59)	0.116	-	-
CKD stage 4-5	2.19 (0.51-9.37)	0.292	-	-
Dementia	2.61 (1.43-4.75)	**0.002**	2.27 (1.20-4.30)	**0.012**
CCI	1.10 (0.98-1.25)	0.116	-	-
Risk factors and treatment for skeletal fragility				
Family history of osteoporosis	1.05 (0.57-1.91)	0.886	-	-
Active smoking	1.29 (0.45-3.71)	0.634	-	-
Alcohol abuse	1.79 (0.16-20.04)	0.636	-	-
Glucocorticoid use	2.05 (0.67-6.32)	0.211	-	-
At least one previous fracture	0.91 (0.54-1.55)	0.736	-	-
At least one previous MOF	0.74 (0.41-1.32)	0.306	-	-
At least one previous HF	0.97 (0.42-2.28)	0.953	-	-
At least one previous vertebral fracture	0.46 (0.19-1.14)	0.095	0.40 (0.16-1.04)	0.060
FRAX MOF (%)*, median (IQR)	0.99 (0.96-1.02)	0.468	-	-

Abbreviations: 95% CI: 95% confidence interval, BMI: body mass index, CCI: Charlson Comorbidity Index, CKD: chronic kidney disease, COPD: chronic obstructive pulmonary disease, FRAX: fracture risk assessment tool, HF: hip fracture, MOF: major osteoporotic fracture, OR: odds ratio, VDD: Vitamin D deficiency. *Calculated not including index hip fracture. Significant p-values are highlighted in bold.

**Table S3. Logistic regression analysis for factors associated with home vitamin D supplementation.**				
	**Univariable analysis**		**Multivariable analysis**	
**Predictor Variable**	**OR (95% CI)**	**p**	**OR (95% CI)**	**p**
Age (10 years)	0.97 (0.83-1.12)	0.651	1.06 (0.89-1.27)	0.515
Female sex	3.35 (2.37-4.73)	**<0.001**	2.95 (2.01-4.33)	**<0.001**
BMI (kg/m^2)	0.96 (0.93-0.99)	**0.009**	0.97 (0.94-1.00)	0.075
Comorbidities				
Hypertension	1.10 (0.82-1.46)	0.523	-	-
Previous myocardial infarction	0.63 (0.40-0.97)	**0.038**	1.11 (0.67-1.83)	0.685
Heart failure	0.59 (0.37-0.94)	**0.025**	0.65 (0.39-1.10)	0.106
Previous venous thromboembolism	1.17 (0.66-2.10)	0.585	-	-
Previous stroke	0.80 (0.50-1.27)	0.345	-	-
Diabetes mellitus	0.87 (0.61-1.24)	0.446	-	-
COPD	0.56 (0.31-0.99)	**0.047**	0.93 (0.49-1.78)	0.833
Rheumatic diseases	3.26 (1.82-5.84)	**<0.001**	2.53 (1.34-4.79)	**0.004**
Liver cirrhosis	2.74 (0.77-9.77)	0.121	-	-
CKD stage 4-5	0.50 (0.23-1.12)	0.092	0.47 (0.18-1.26)	0.135
Dementia	0.63 (0.45-0.88)	**0.006**	0.57 (0.39-0.83)	**0.003**
CCI	0.95 (0.89-1.02)	0.134	-	-
Risk factors and treatment for skeletal fragility				
Family history of osteoporosis	1.68 (1.21-2.35)	**0.002**	1.60 (1.10-2.32)	**0.015**
Active smoking	0.60 (0.35-1.03)	0.064	0.72 (0.39-1.34)	0.303
Alcohol abuse	0.36 (0.10-1.24)	0.104	-	-
Glucocorticoid use	2.59 (1.14-5.91)	**0.023**	2.07 (0.82-5.26)	0.124
At least one previous fracture	1.84 (1.39-2.44)	**<0.001**	1.69 (1.23-2.31)	**0.001**
At least one previous MOF	1.87 (1.38-2.55)	**<0.001**	-	-
At least one previous HF	1.42 (0.87-2.30)	0.160	-	-
At least one previous vertebral fracture	1.82 (1.19-2.76)	**0.005**	-	-
FRAX MOF (%)*, median (IQR)	1.04 (1.03-1.06)	**<0.001**	-	-

Abbreviations: 95% CI: 95% confidence interval, BMI: body mass index, CCI: Charlson Comorbidity Index, CKD: chronic kidney disease, COPD: chronic obstructive pulmonary disease, FRAX: fracture risk assessment tool, HF: hip fracture, MOF: major osteoporotic fracture, OR: odds ratio, VDD: Vitamin D deficiency. *Calculated not including index hip fracture. Significant p-values are highlighted in bold.

**Table S4. Baseline characteristics of patients with and without 12-month follow-up data.**			
	**No 12-month follow-up available** (n = 380)	**12-month follow-up available** (n = 554)	**p**
Age (year), mean (SD)	82.7 (8.73)	83.1 (9.02)	0.559
Female sex, n (%)	274 (72)	391 (71)	0.659
BMI (kg/m^2), mean (SD)	24.0 (4.27)	24.3 (4.51)	0.258
Comorbidities			
Hypertension, n (%)	261 (69)	369 (67)	0.523
Previous myocardial infarction, n (%)	46 (12)	66 (12)	0.919
Heart failure, n (%)	41 (11)	64 (12)	0.753
Previous venous thromboembolism, n (%)	20 (5)	31 (6)	0.884
Previous stroke, n (%)	35 (9)	55 (10)	0.736
Diabetes mellitus, n (%)	63 (17)	109 (20)	0.264
COPD, n (%)	23 (6)	43 (8)	0.364
Rheumatic diseases, n (%)	22 (6)	29 (5)	0.770
Liver cirrhosis, n (%)	3 (1)	7 (1)	0.748
CKD stage 4-5, n (%)	12 (3)	24 (4)	0.392
Dementia, n (%)	93 (24)	129 (23)	0.696
CCI, mean (SD)	5.3 (2.0)	5.5 (2.0)	0.618
Risk factors and treatment for skeletal fragility			
Family history of osteoporosis, n (%)	77 (20)	98 (18)	0.348
Active smoking, n (%)	34 (9)	40 (7)	0.388
Alcohol abuse, n (%)	7 (2)	11 (2)	1.000
Glucocorticoid use, n (%)	8 (2)	16 (3)	0.532
At least one previous fracture, n (%)	88 (23)	217 (39)	**<0.001**
At least one previous MOF, n (%)	65 (17)	154 (28)	**<0.001**
At least one previous HF, n (%)	23 (6)	32 (6)	0.915
At least one previous vertebral fracture, n (%)	16 (4)	83 (15)	**<0.001**
FRAX MOF (%)*, median (IQR)	19.0 (10.0-24.0)	20.0 (10.0-29.0)	0.222
Previous vitamin D supplements, n (%)	130 (34)	203 (37)	0.487
Previous anti-fracture treatment, n (%)	32 (8)	46 (8)	1.000

Abbreviations: BMI: body mass index, CCI: Charlson Comorbidity Index, CKD: chronic kidney disease, COPD: chronic obstructive pulmonary disease, FRAX: fracture risk assessment tool, HF: hip fracture, IQR: interquartile range, MOF: major osteoporotic fracture, SD: standard deviation. *Calculated not including index hip fracture. Significant p-values are highlighted in bold.

Figure S1. Correlation matrix of laboratory tests.

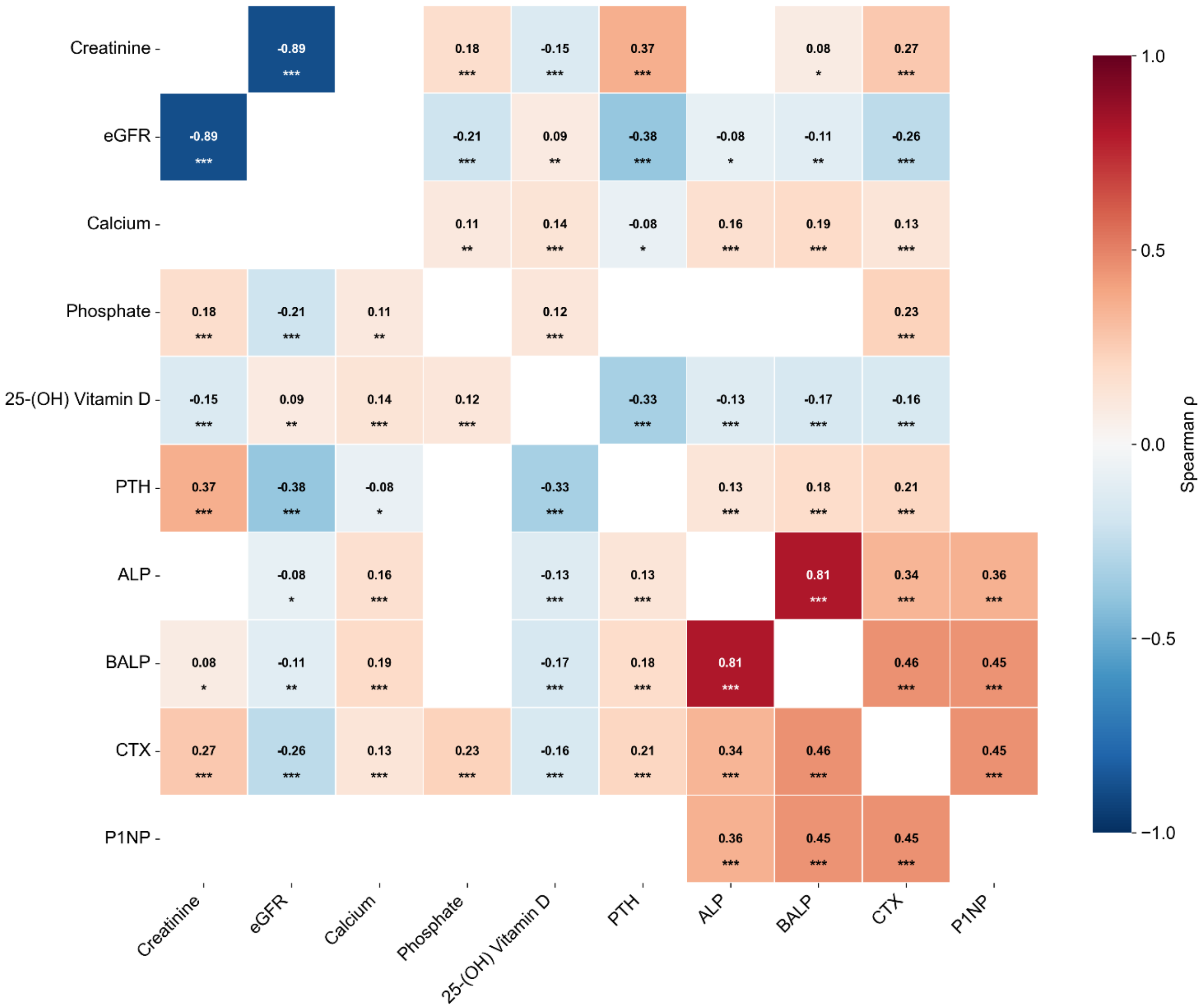

Spearman correlation coefficients between laboratory tests in the study cohort. Correlations with p≥0.05 are not displayed. *p<0.05, **p<0.01, ***p<0.001.

Figure S2. 12-month survival by baseline 25-(OH)D levels in patients without (A) and with prior vitamin D supplementation (B).

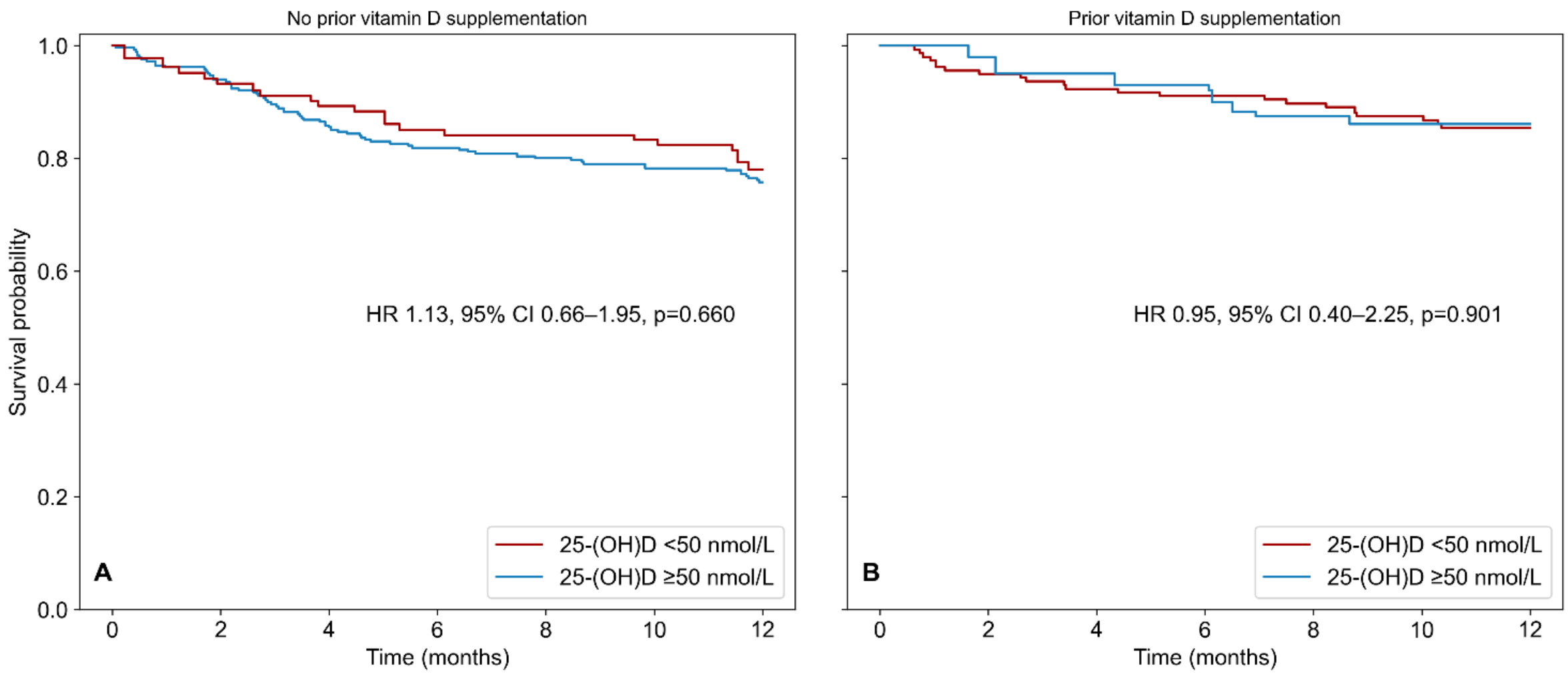

Abbreviations: 25-(OH)D: 25-(OH) vitamin D, 95% CI: 95% confidence interval, HR: hazard ratio.
